# Highly ordered ultralong magnetic nanowires wrapped in stacked graphene layers

**DOI:** 10.3762/bjnano.3.95

**Published:** 2012-12-11

**Authors:** Abdel-Aziz El Mel, Jean-Luc Duvail, Eric Gautron, Wei Xu, Chang-Hwan Choi, Benoit Angleraud, Agnès Granier, Pierre-Yves Tessier

**Affiliations:** 1 Institut des Matériaux Jean Rouxel, IMN, Université de Nantes, CNRS, 2 rue de la Houssinière, BP 32229, 44322 Nantes cedex 3, France, Telephone: +33 (0) 240 376 434, Fax: +33 (0) 240 373 959; 2 Department of Mechanical Engineering, Stevens Institute of Technology, Hoboken, NJ 07030, USA

**Keywords:** carbon, ferromagnetic, graphene, nanofabrication, nanowires, nickel, phase separation

## Abstract

We report on the synthesis and magnetic characterization of ultralong (1 cm) arrays of highly ordered coaxial nanowires with nickel cores and graphene stacking shells (also known as metal-filled carbon nanotubes). Carbon-containing nickel nanowires are first grown on a nanograted surface by magnetron sputtering. Then, a post-annealing treatment favors the metal-catalyzed crystallization of carbon into stacked graphene layers rolled around the nickel cores. The observed uniaxial magnetic anisotropy field oriented along the nanowire axis is an indication that the shape anisotropy dominates the dipolar coupling between the wires. We further show that the thermal treatment induces a decrease in the coercivity of the nanowire arrays. This reflects an enhancement of the quality of the nickel nanowires after annealing attributed to a decrease of the roughness of the nickel surface and to a reduction of the defect density. This new type of graphene–ferromagnetic-metal nanowire appears to be an interesting building block for spintronic applications.

## Introduction

Magnetic nanowires have been widely investigated during the last two decades for fundamental physics [1–7], and nano-engineering [7–10]. The various properties of these nanostructures make them very interesting as building block materials for applications in spintronics [8,11], nanobiotechnology [9–10], and for the development of magnetic storage media [12]. Despite a long history of study devoted to the development of different fabrication strategies with a predilection for template methods [1–12], ferromagnetic nanowires still suffer from their relatively short length, which cannot reach up to the macroscopic scale. In addition, the manipulation of such one-dimensional (1D) nanostructures is often considered as a complicated process and a barrier for a simple integration of nanowires into electrical devices.

The past few years have witnessed the rise of graphene as an extraordinary functional material with unique properties [13–16]. This material is one of the best candidates that can be used for the development of electronics, sensors, and energy-related devices [17–19]. The combination between the electrical properties of graphene and the magnetic properties of 1D ferromagnetic nanostructures would offer wide prospects for spintronic applications. Fabricating coaxial nanowires with ferromagnetic cores and graphene stacking shells is an elegant way to combine the unique properties of these two materials [20–23]. In addition, similar to a polymeric layer covering a magnetic nanowire [7], and considering the efficient protection of a single graphene layer [24], the stacked graphene layers (i.e., the shell) wrapping the nanowires could be considered as an outstanding shield protecting the metal cores against oxidation. Core–shell nanowires consisting of metal cores and graphene stacking shells, also known as metal-filled carbon nanotubes, are in general produced by chemical vapor deposition (CVD) [20–23]. Such a technique allows accurate controlling over the characteristics (i.e., density, length, tube diameter, etc.) of the vertically grown metal-filled nanotubes. Despite this accurate growth control, CVD does not allow the growth of metal-filled nanotubes with a length up to the macroscale while retaining an excellent alignment. Additionally, in some cases the metal is found to be discontinuous inside the tubes [20].

**Figure 1:**
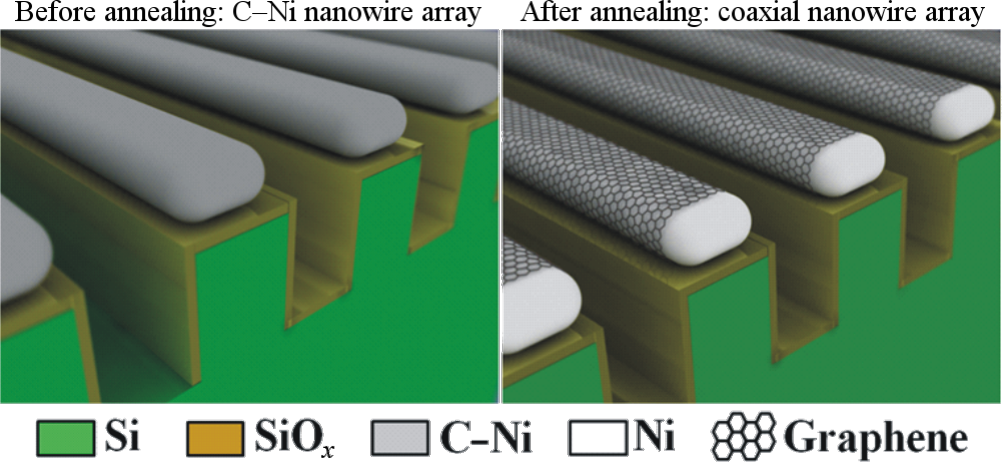
Schematics of the carbon-containing nickel nanowire array before (left) and after (right) post-annealing for 1 h at 400 °C.

In a previous study, we demonstrated the possibility to synthesize an array of aligned Ni nanowires on a patterned silicon surface [25]. In this letter we present a simple and efficient method to prepare an array of highly ordered coaxial nickel/graphene-stacks core–shell nanowires with a length up to 1 cm. The process involves the deposition of nickel nanowires containing a low amount of carbon (3 atom %) by a hybrid sputtering technique [26] on a patterned silicon substrate consisting of periodic nanograting structures (Figure 1, left) prepared by laser interference lithography coupled to deep reactive-ion etching [27]. After the deposition, in order to form stacked graphene layers rolled around the nickel nanowires (Figure 1, right), the carbon-containing nickel (C–Ni) nanowires were thermally annealed at 400 °C for 60 min (details concerning the selection of the annealing conditions are presented in Supporting Information File 1).

## Results and Discussion

**Figure 2:**
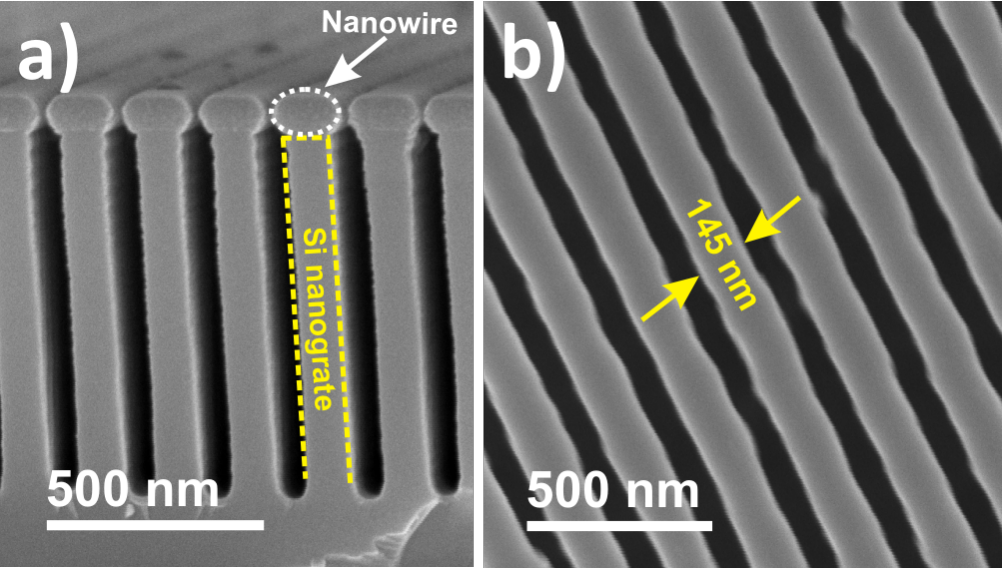
SEM micrographs of the post-annealed carbon-containing nickel nanowires on silicon nanograted structures. Cross section (a) and plan (b) view.

The SEM micrographs of the post-annealed carbon-containing nickel nanowires (Figure 2) show the organization and alignment of these nanostructures on the top surface of the silicon nanogrates. The nanowires have a homogenous morphology with a mean diameter of ≈145 nm (corresponding to the width of the grates) and a length up to 1 cm (corresponding to the size of the substrate). The preferential growth of nickel on the top surface of the grating structures can be mainly attributed to the following mechanisms: (i) the low directionality of the deposition process; (ii) the low width of the nanotrench separating two subsequent nanogrates; and (iii) the high depth-to-width ratio of the trenches (here, typically 12) [25,28].

**Figure 3:**
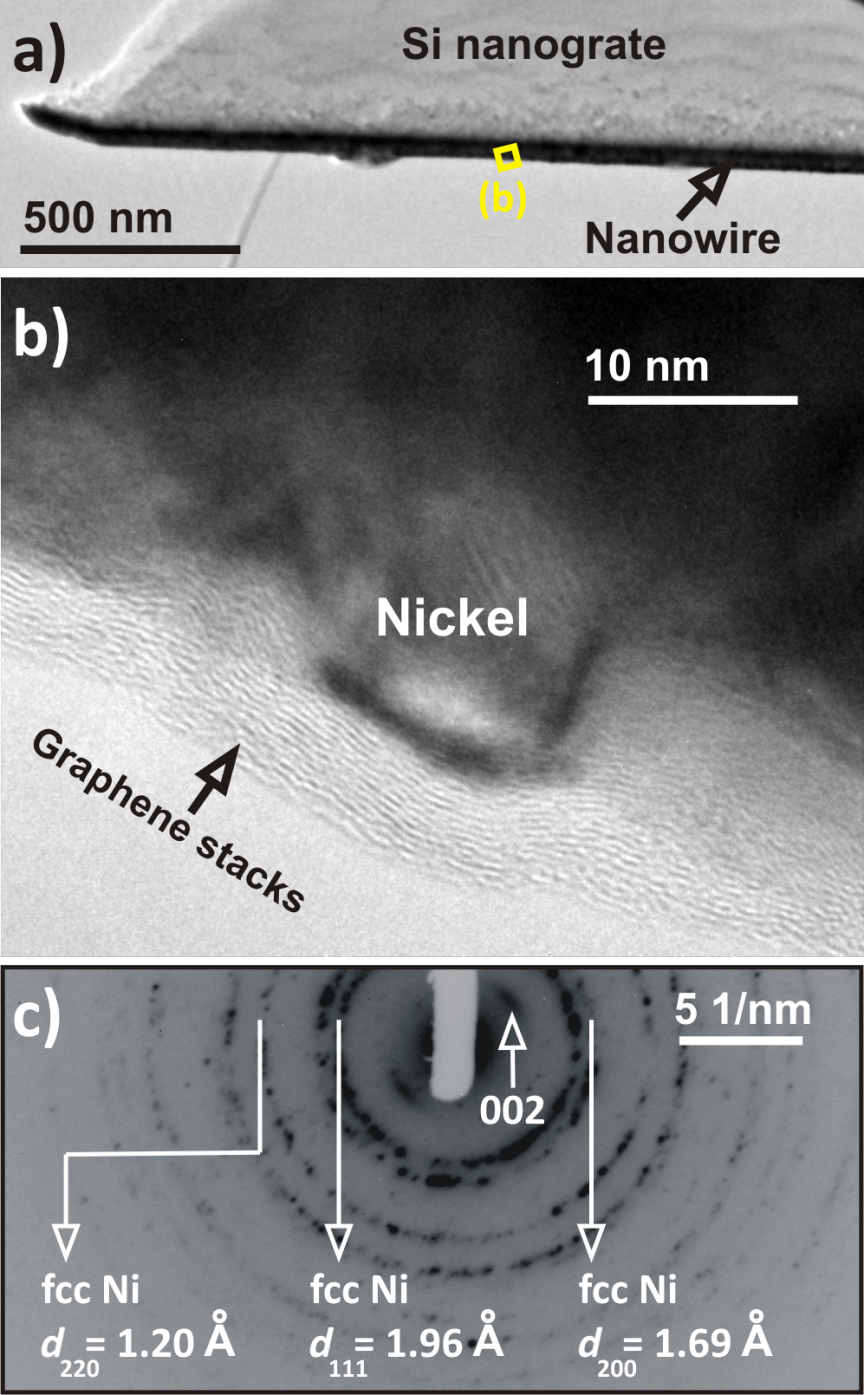
(a) TEM micrograph of a coaxial nanowire as prepared on a silicon nanograted structure. (b) High-resolution TEM micrograph showing the presence of several stacked graphene layers wrapping the nickel nanowire. (c) Selected-area electron diffraction pattern recorded on a single wire. The 002 reflection indicated in (c) is attributed to graphitic carbon.

In order to prove that these nanowires have a core–shell structure with a nickel core and graphene stacking shell, they were placed on a carbon-coated copper grid and their surfaces were examined by TEM (Figure 3a). A typical high-resolution TEM micrograph of the surface of a nanowire is presented in Figure 3b. The TEM analysis reveals the presence of a few graphene stacked layers (ca. 12) with a low nanotextural order neighboring the nickel nanowire surface. The interlayer distance of two adjacent graphene layers, evaluated from the high-resolution TEM micrograph, was about 0.347 nm. This value is very close to the interlayer distance of two graphene monolayers in graphite (0.335 nm). The presence of the graphene stacks was further demonstrated by electron diffraction (Figure 3c). The obtained diffraction pattern was very similar to the one recorded on Ni-filled carbon nanotubes that we synthesized in a previous study by thermal annealing of Ni nanowires organized in an amorphous carbon film [29]. Thus, although the synthesis method developed in this work is completely different to the one used in our previous study [29], the nanostructures obtained with both methods exhibit a similar crystalline structure.

Indeed, in both cases only the 002 reflections corresponding to crystalline hexagonal graphite were present [29–30]. They appear as arcs instead of rings due to the anisotropic nanotexture in the analyzed area [30]. The 004 reflections and *hk* bands (i.e., 10 and 11 bands of turbostratic carbon) are probably absent due to the low number of analyzed stacked graphene layers. The other rings observed on the diffraction pattern are attributed to face-centered cubic (fcc) nickel. The formation of the stacked graphene layers results from the phase separation and the nickel-catalyzed crystallization of carbon by thermal annealing. During the post-annealing stage of the carbon-containing nickel nanowires, the carbon atoms diffuse and homogenously dissolve in the nickel phase [31–32]. As the limit of the solid solubility of carbon in the nickel phase is reached during the cool-down step, the carbon atoms precipitate into graphene stacked layers on the free surface of the nanowires. This mechanism has been recently used for the synthesis of a few layered graphene sheets [31,33–34].

**Figure 4:**
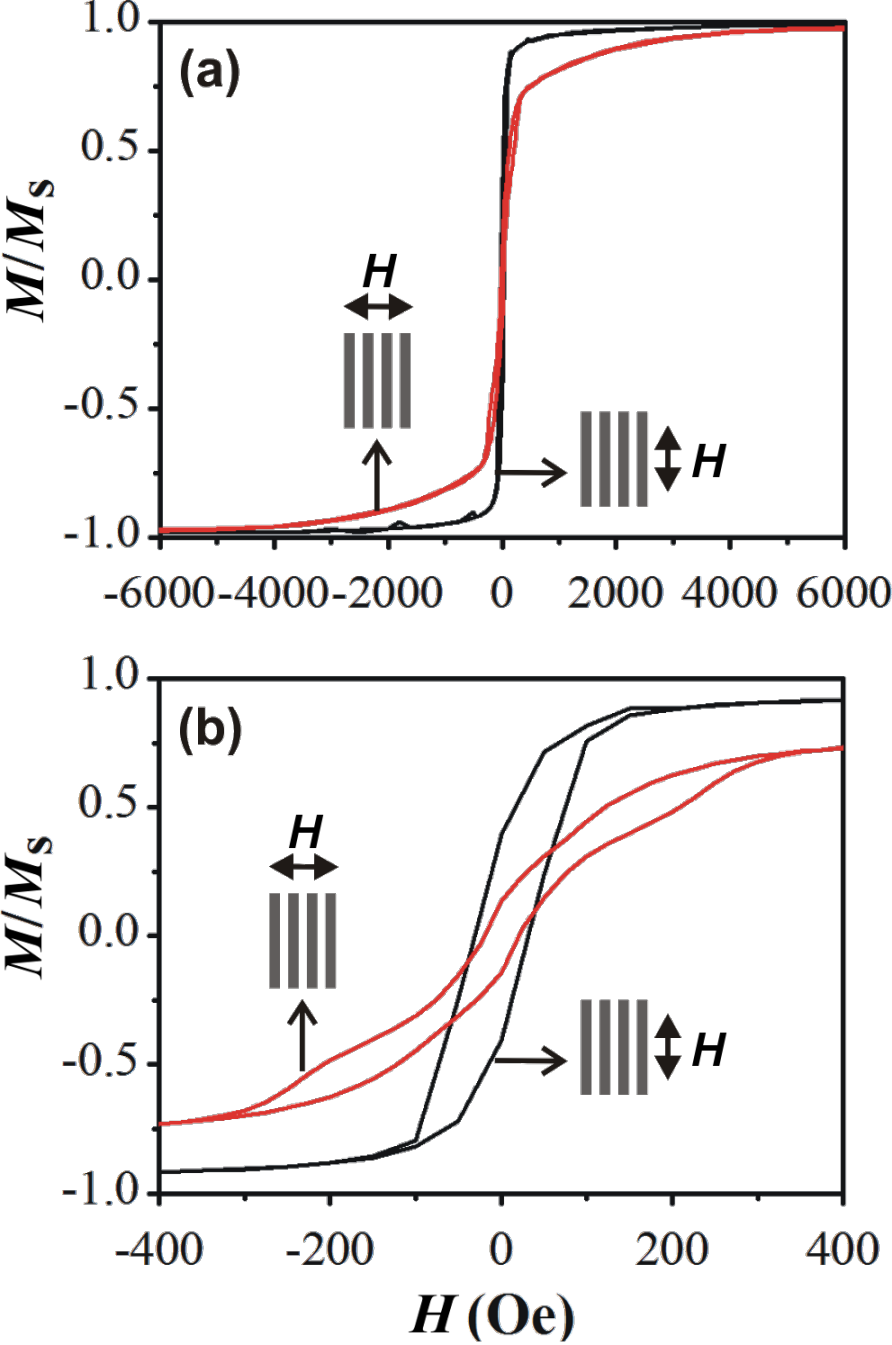
(a) Normalized hysteresis loops of the coaxial nanowire array measured at 300 K with an applied magnetic field parallel (black curve) and perpendicular (red curve) to the wire axis. Panel (b) is a magnified region of (a).

The magnetic behavior of the nanowire arrays after post-annealing has been investigated at 300 K by using a Quantum Design SQUID magnetometer. The in-plane magnetization hysteresis loops were measured for an applied field parallel (black curve) and perpendicular (red curve) to the wire axis (Figure 4). The saturation fields, measured in both configurations, were found to be almost equal to the ones obtained for the as-grown C–Ni nanowires before annealing (Table 1 and Supporting Information File 1, Figure S3). The smaller saturation field (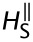 = 1500 Oe) and the larger squareness (*M*_r_/*M*_s_ = 0.4) when the external magnetic field is applied parallel to the nanowire axis, compared to the perpendicular configuration (roughly 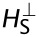 = 3100 Oe and *M*_r_/*M*_s_ = 0.14), indicate that the nanowire array exhibits a preferential magnetic orientation along the wire axis (i.e., easy axis parallel to the nanowires).

**Table 1:** Summary of the magnetic characteristics recorded at 300 K for C–Ni nanowire arrays before and after thermal annealing at 400 °C.

Sample	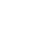 (Oe)	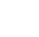 (Oe)	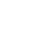 (Oe)	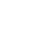 (Oe)

As-grown C–Ni nanowires	1550	3100	127	34
Postannealed C–Ni nanowires	1500	3100	32	21

In the case of polycrystalline ferromagnetic nanowires, such uniaxial magnetic anisotropy originates from the shape anisotropy resulting from the very high aspect ratio of these nanostructures [7,35–43]. Concerning the coercive field, it is slightly higher (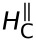 = 32 Oe) when the external magnetic field is applied parallel to the nanowire array, than the one measured for the perpendicular configuration (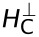
= 21 Oe). It can be seen that the measured values are very low compared to the ones reported in literature for nickel nanowires with the same diameter (roughly 350 Oe) [3].

Here, for nickel nanowires with a 150 nm diameter, a multidomain configuration is expected. Indeed, this diameter is significantly larger than the exchange length λ_ex_ and the domain wall width λ_w_ whose values for nickel are about 20 and 90 nm, respectively [44]. Moreover, the small gap of about 100 nm between two subsequent nanowires can promote dipolar coupling between the wires. Indeed, the magnetic interactions between separated lines are due to magnetostatic effects that become relevant when the line separation is typically less than the line width [38,42]. For a field applied parallel to the lines, the coercive field and the squareness are reduced when the line separation decreases [36,38,41]. As a consequence, no square hysteresis loop, as measured for single-domain and isolated nanowires [3], is expected here, and complex magnetic configurations can take place. Moreover, by comparing the coercive fields measured before and after the post-annealing procedure we can conclude that they are reduced after thermal annealing (Table 1). The decrease in the coercive fields suggests that the surface of the nickel wire, i.e., the interface with the graphene shell, becomes very smooth after annealing since morphological defects favor pinning of the domain walls and, thus, result in higher coercivity [34].

The presence of carbon impurities within the as-grown C–Ni nanowires may also be another factor resulting in higher coercive fields before annealing, since these impurities may generate some defects in the crystalline structure of the nickel phase. In addition, the small coercive fields recorded after annealing suggest that there is no oxide layer surrounding the nickel core, which would produce a bias exchange between the magnetizations of nickel and nickel oxide. This non-oxidized nickel nanowire is expected due to the presence of the stacked graphene layers as a barrier.

## Conclusion

In summary, an efficient method for the synthesis of an array of ultralong and organized coaxial nanowires, with nickel cores and graphene stacking shells, has been demonstrated. The TEM analysis revealed that the stacked graphene layers forming the shell have a turbostratic structure and a nanotextural order. We have further demonstrated the presence of a preferential magnetic orientation along the wire axis, which has been attributed to the shape anisotropy. The low coercive fields reflect the low roughness and low structural defects as well as dipolar coupling between the nanowires. This new type of graphene ferromagnetic metal nanowire appears to be an interesting building block for spintronics, for example, for the injection of a spin-polarized current from the metal to the high-carrier-mobility graphene structure. Its integration in a planar configuration opens the way to further device characterization. Moreover, the metal-catalyzed crystallization of carbon by thermal annealing, which is the mechanism employed in this work, allows the synthesis of graphene sheets of a few layers with low defects [31–33]. Therefore, after an optimization of the materials and the processing conditions of the technique developed in this work (e.g., the amount of carbon incorporated in the nickel phase, the post-annealing temperature, the metal used as catalyst, the dimensions of the nanograting structures, etc.) this strategy can be adopted for the growth of graphene nanoribbons a few layers thick and of macroscopic length.

## Experimental

As described elsewhere [27], the nanograted substrate, which served as a template to prepare the nanowires, was fabricated by laser interference lithography followed by deep reactive ion etching. The size of the substrate was 1 × 1 cm^2^, and the periodicity of the nanograting patterns was 240 nm. The width of each nanograte was about 140 nm. The details of the plasma process employed for the synthesis of the (C–Ni) nanowires are presented elsewhere [26]. Briefly, it consists of simultaneous depositions of metal and carbon by using a hybrid sputtering technique. For the deposition of nickel, a radio-frequency (RF) generator, operating at 13.56 MHz, was connected to a magnetron source in order to sputter a nickel target of 50 mm in diameter and 99.99 % in purity. For the simultaneous deposition of carbon, a carbon-coated one-turn stainless coil was placed at equal distance between the nickel target and the substrate. When applying RF power of 150 W to this coil, an additional plasma of pure argon was generated leading to the sputtering of the carbon layer coated on the coil, and hence, a small amount of carbon (3 atom %) was deposited. The base pressure before deposition was 10^−4^ Pa, whereas the deposition argon pressure was fixed to 0.67 Pa. The deposition was performed for 2 min at a floating potential and at a low temperature (*T* < 150 °C). After the growth, the postannealing treatment of the C–Ni nanowires at 400 °C for 60 min was performed in an oven at atmospheric pressure and under argon flow. After annealing, the samples were cooled down at a rate of 12 °C/min. Scanning electron microscopy (SEM) imaging was performed at 5 kV on a JEOL JSM 7600 F microscope. Transmission electron microscopy (TEM) imaging and selected-area electron diffraction (SAED) were performed on a Hitachi H-9000 NAR microscope (LaB_6_ filament, 300 kV, Scherzer resolution: 0.18 nm). After the postannealing procedure of the carbon-containing Ni nanowires, the TEM specimens were prepared by a simple scratching of the sample surface with a pair of tweezers over a carbon-coated copper grid. Then, a drop of ethanol was placed on to the copper grid for the purpose of dispersion of the collected nanostructures.

## Supporting Information

The optimization of the thermal-annealing procedure and the magnetic characterization of the as-grown nanowires are available in the Supporting Information.

File 1 Annealing procedure and hysteresis loops of the as-grown C–Ni nanowires.
