# Supplementary material for: Highly ordered ultralong magnetic nanowires wrapped in stacked graphene layers
Source: Beilstein J Nanotechnol. 2012 Dec 11;3:846–51. doi: 10.3762/bjnano.3.95 (PMC3556984; doi:10.3762/bjnano.3.95)
Supplement: File 1 — Annealing procedure and hysteresis loops of the as-grown C–Ni nanowires. [file Beilstein_J_Nanotechnol-03-846-s001.pdf]

Supporting Information

for

Highly ordered ultralong magnetic nanowires wrapped in stacked graphene layers

Abdel-Aziz El Mel[1], Jean-Luc Duvail[1], Eric Gautron[1], Wei Xu[2], Chang-Hwan Choi[2], Benoit Angleraud[1], Agnès Granier[1] and Pierre-Yves Tessier[1*]

Address: [1]Institut des Matériaux Jean Rouxel, IMN, Université de Nantes, CNRS, 2 rue de la Houssinière, BP 32229, 44322 Nantes cedex 3, France, Telephone: +33 (0) 240 376 434, Fax: +33 (0) 240 373 959 and [2]Department of Mechanical Engineering, Stevens Institute of Technology, Hoboken, NJ 07030, USA

*Corresponding author

E-mail: Pierre-Yves Tessier - Pierre-Yves.Tessier@cnrs-imn.fr

Annealing procedure and hysteresis loops of the as-grown C–Ni nanowires

1. Optimization of the annealing temperature

In order to investigate the effect of the post-annealing temperature, the carbon-containing nickel nanowires were annealed at three different temperatures. The sample post-annealed at 400 °C (i.e., results presented in the article) showed a homogenous and smooth morphology very similar to the one before annealing (Figure S1a). It was observed that at a higher temperature (600 °C) a morphological modification occurs and the surface roughness of the nanowires increases (Figure S1b). Upon reaching 800 °C, discontinuous nanochains appeared instead of nanowires (Figures S1c and S1d). The morphological modification as a function of the post-annealing temperature was confirmed by TEM (Figure S2). The nanowires postannealed at 600 °C show rough surfaces. In addition, nickel nanoparticles were observed on the sidewall of the nanograting structures probably resulting from the nickel phase diffusion (Figures S2a and S2b). The high roughness is not desirable for the growth of continuous stacked graphene layers, whereas the nickel nanoparticles formed on the sidewall of the nanogrates (Figure S2c) could disturb the magnetic measurements performed on the nanowires. Upon reaching 800 °C, the nanowires are no longer continuous, and nickel nanochains, consisting of sets of linked nanoparticles, were formed (Figures S2c and S2d).

Based on the results obtained in this study, we can conclude that an appropriate temperature to form coaxial nanowires, with nickel cores and graphene stacking shells, should be less than 600 °C. This is the main reason why the postannealing temperature was selected to be 400 °C in the study presented in the article.

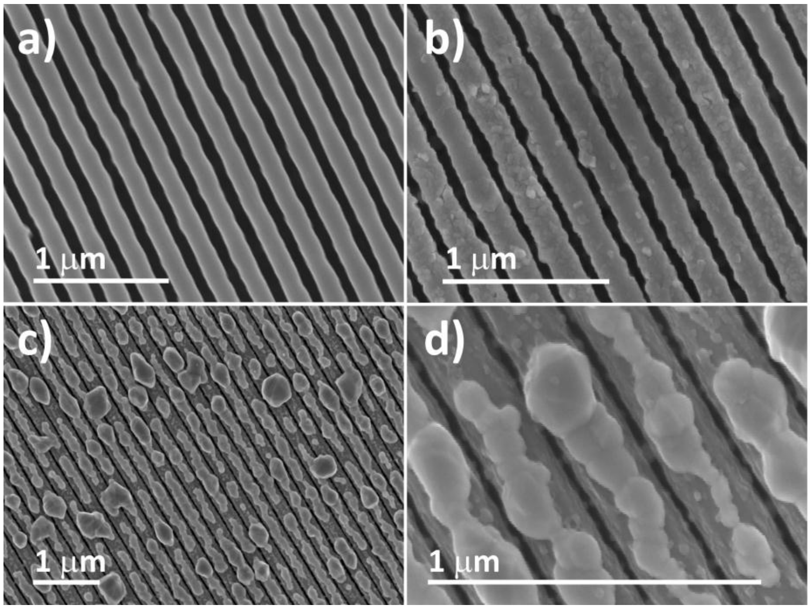

Figure S1: SEM images of carbon-containing nickel nanowires postannealed at: (a) 400 °C, (b) 600 °C, and (c, d) 800 °C.

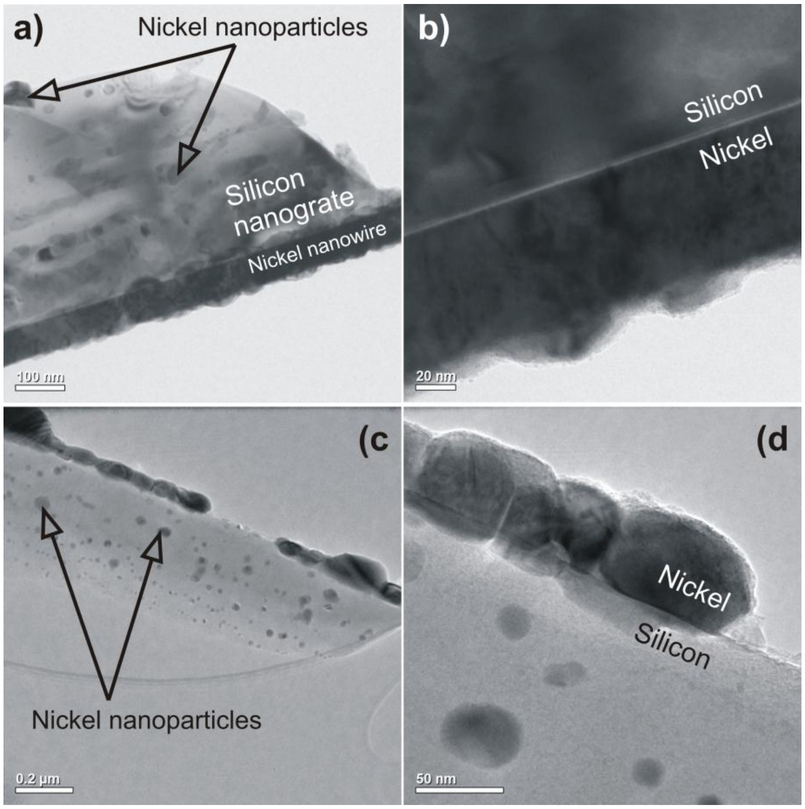

Figure S2: TEM micrographs of carbon-containing nickel nanowires post-annealed at: (a, b) 600 °C and (c, d) 800 °C.

2. Magnetic characterization of as-grown C–Ni nanowires

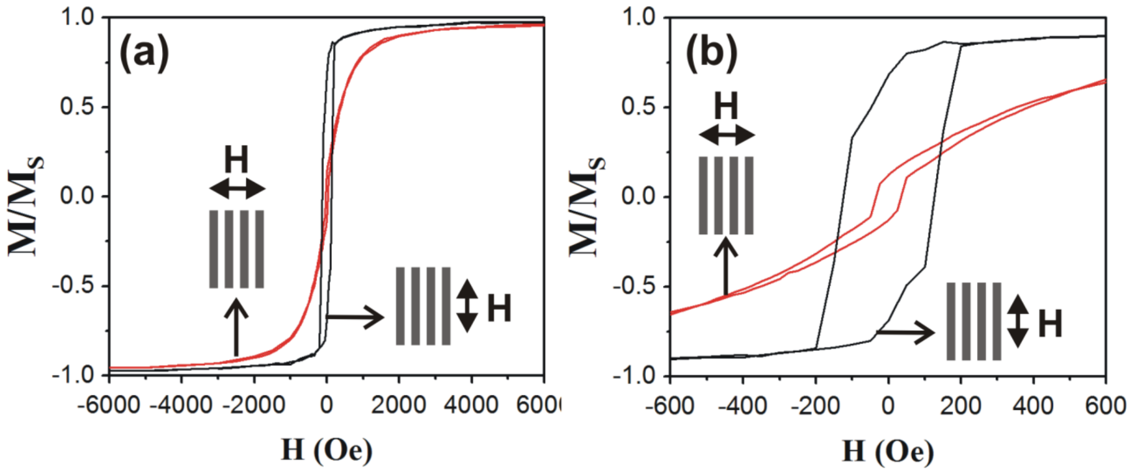

Figure S3: (a) Normalized hysteresis loops of carbon-containing nickel nanowire array before annealing measured at 300 K with an applied magnetic field parallel (black curve) and perpendicular (red curve) to the wire axis. Panel (b) is a magnified region of (a).
